# Do world-wide policy initiatives for regulating health care related artificial intelligence safeguard the declaration of Helsinki?

**DOI:** 10.1016/j.eclinm.2026.103784

**Published:** 2026-02-11

**Authors:** Antonis A. Armoundas, Joseph Loscalzo

**Affiliations:** a Cardiovascular Research Center, Massachusetts General Hospital, United States; b Broad Institute, Massachusetts Institute of Technology, United States; c Division of Cardiovascular Medicine, Channing Division of Network Medicine, Department of Medicine, Brigham and Women's Hospital, Harvard Medical School, United States

**Keywords:** Artificial intelligence, Policy, Regulation, Global, Human rights

## Abstract

Digital health technologies and artificial intelligence (AI), are transforming medical research, health care, and public health. The ever-increasing usage of algorithms in health care has challenged governments, regulatory agencies, health organizations, developers, and providers, and AI raises novel ethical challenges that extend beyond the jurisdiction of traditional borders and regulatory health–care processes and structures. While there is growing consensus in recognizing these ethical challenges, there is less agreement over the necessary AI guardrails. This Viewpoint offers a synthesis of representative AI-enabled health policy approaches across jurisdictions and advances practical recommendations for an adaptive, international AI policy and governance framework that will be responsible for monitoring and advancing its regulations in pace with the rapid growth of AI technologies. We use the Declaration of Helsinki as a normative reference point, to derive risk-proportionate safeguards for AI-enabled health across research and non-research settings.

## Introduction

The Declaration of Helsinki (DoH) is a living statement of principles, including patient rights, such as dignity, informed consent, privacy and confidentiality, intended to guide the ethical conduct of global health and medical research, developed and endorsed by the World Medical Association (WMA).^1^ The WMA holds that these principles should be upheld by all individuals, teams, and organizations involved in medical research, considered as fundamental for the respect and protection of all research participants, both patients and healthy volunteers.

As digital health technologies^2^ and artificial intelligence (AI)^3^ are transforming medical research, health care, and public health, these ethical challenges must be adequately addressed, if AI is to be widely adopted and used to improve medicine and human health, to preserve and enhance human autonomy,^4^ and to safeguard equitable access to these technologies.^5^^,^^6^

Recently, governments have taken action in exploring and enacting national policies for AI research, development, deployment, and most importantly, use in many diverse areas, including healthcare. These initiatives reflect their substantive priorities, and, commonly, they also recognize the need for responsible conduct in innovation that is grounded in universal human rights, as well as the country's national values. While there is growing consensus in recognizing these ethical challenges, there is less agreement over the necessary AI guardrails, that safeguard the modernized ethical principles aiming to promote and ensure respect for all participants.^7^

In this Viewpoint, we have considered major regulatory bodies like the United Nations and the European Union, as well as countries, worldwide, listed at the top in applying artificial intelligence in the life science research (including publications, quality-adjusted-productivity and relevance),^8^ such as the United States, China, the United Kingdom, India, Canada and Japan. This Viewpoint purposively presents an interpretive synthesis, rather than a systematic and exhaustive search, of policies of selected authoritative, international public-policy bodies and jurisdictions with global influence or distinct approaches to AI-enabled health, and proposes tangible recommendations for an international framework to oversee these regulations and policies,^9^ in accordance with the DoH (Box 1). DoH is used as a principled baseline to translate into proportionate safeguards for high-impact health-AI, and not to expand research-ethics law to all AI.

Box 1 **The 2024 revisions of the DoH associated with artificial intelligence.**

**Table:** 

Data & model training/secondary use	New paragraph 32 requires free, informed consent for the collection, processing, storage, and foreseeable secondary use of biological material and identifiable or re-identifiable data; databases/biobanks must be Research Ethics Committee (REC)-approved and monitored. The text explicitly cross-references the World Medical Association’s Declaration of Taipei, which governs health databases/biobanks (and extends beyond research), making it the companion framework for artificial intelligence (AI)-enabled data pipelines.
Independent oversight for AI evaluations	Revised REC provisions (paragraph 23) mandate committee independence, resources, local context expertise, public membership, authority to monitor/suspend, approval in both sponsor and host countries, and a final report, all directly applicable to AI trials, registries, and real-world performance studies.
Continuous risk–benefit management	Paragraphs 16–18 require ongoing monitoring with obligations to modify or stop studies when risks outweigh the benefits or the outcomes are conclusive, well suited to adaptive/learning AI algorithms whose performance can drift.
Registration and full reporting	Paragraphs 35–36 require prospective public registration, as well as making all results (including negative/inconclusive) available, creating guardrails against selective reporting or hype in AI studies.
Consent & participant rights	Modernized consent (paragraphs 25–31) emphasizes plain-language information, attention to dependent relationships, options for participants to receive general study results, and defined emergency pathways, relevant when deploying decision-support AI in acute care environments, or when models use identifiable data.
Equity, inclusion, and vulnerability	Paragraphs 19–20 reframe vulnerability as contextual/dynamic and require weighing harms of exclusion vs inclusion, a basis for sampling plans and subgroup performance checks to mitigate AI bias.
Unproven interventions in clinical practice	Paragraph 37 (unchanged in spirit, but clarified) covers the ethically constrained use of unproven interventions of clinical trials, with duties to seek expert advice, obtain consent, record/share data, and avoid compromising trials, a direct analog for the early AI use at point of care.
Scope & responsibility broadened	The Preamble now calls on all individuals, teams, and organizations (not only physicians) to uphold DoH principles, capturing multidisciplinary AI teams.
Environmental sustainability	Paragraph 11 adds a duty to avoid/minimize environmental harm, which is increasingly relevant to computing-intensive AI.

## Applicability to healthcare and commercial health

This Viewpoint distinguishes (i) health-specific regimes (e.g., health-privacy laws; medical-device regulation for software/AI) from (ii) general AI frameworks that apply to health uses based on function and risk. In the United States, the Health Insurance Portability and Accountability Act (HIPAA) applies to covered entities (e.g., health plans, certain providers, etc) and their business associates. Many commercial entities that are handling health-related information (e.g., some consumer health apps, wearables platforms, etc) are not HIPAA-covered, even when data are clinically sensitive (Box 2). Their obligations arise instead from general consumer-protection and privacy laws (e.g., prohibitions on unfair or deceptive practices, breach-notification, and state health-privacy statutes).

Box 2 **AI policy clinical translation to healthcare and commercial health.**

**Table:** 

Health care delivery	Governance begins with health-privacy/confidentiality rules and responsibilities: when an AI system is used with an intended medical purpose (diagnosis, prevention, treatment, mitigation), medical-device law applies to the product regardless of who deploys it, while data-protection law governs the handling of patient information. If the deployment constitutes research or “horizontal” AI frameworks layer on the top of where function/risk thresholds are met (e.g., documentation, transparency, risk management, post-deployment monitoring).
Medical-device AI (SaMD/MDDS)	Obligations are product-centered: premarket evidence of safety/effectiveness should be appropriate to risk; quality management and change–control processes should exist for learning systems; real-world performance monitoring and incident reporting should be documented; and field-corrective actions should be taken when needed. These product responsibilities coexist with data-protection requirements for the datasets used to train, validate, and update models. Whereas there is a jurisdiction of an AI act, additional responsibilities (e.g., risk-management systems, human-factor safeguards), are typically imposed on device rules.
Commercial health outside HIPAA (U.S.)	Many consumer apps, platforms, etc, are not HIPAA-covered, even when handling clinically sensitive information: their baseline obligations arise from consumer-protection and privacy laws (including state health-data statutes), breach-notification responsibilities, and any applicable horizontal AI requirements; if they make medical claims, they may trigger device regulation. Data flows between HIPAA and non-HIPAA entities hinge on business-associate status and contract terms; misleading “HIPAA-compliant” marketing can be deceptive. Good practice therefore includes plain and transparent language, context-appropriate authorization/consent, contract-level limits on data reuse, auditability, and equity checks on model performance.

SaMD: Software as a Medical Device. MDDS: Medical Device Data Systems (an FDA term); it refers to hardware/software **that** transfers, stores, converts format, or displays medical device data without controlling or altering the device's functions or being used for real-time, active patient monitoring. In FDA rules it's a Class I device type (21 CFR 880.6310), generally low-risk and often exempt from premarket notification, though still subject to basic safety/quality requirements.

By contrast, medical-device rules (e.g., for software/AI used for diagnosis, cure, mitigation, treatment, or prevention) are based on intended use, regardless of HIPAA status. In the EU and other jurisdictions, general data-protection law^10^ and sectoral device rules interact with horizontal AI acts or guidance; throughout the paper we state explicitly whether each policy is health-specific or general, and how it applies to care delivery, research, and commercial health contexts.

## Where do we have consensus

An identifiable pattern across AI-governance structures has to do with the different functionalities of AI.^9^ Broadly, three operations can be identified: *first*, constraint, which typically places limits on the development and use of AI; *second*, enabling, which permits or promotes the research, development, and use of AI; *third*, leveling, which includes transparency and disclosure obligations.

A second pattern lies in the diverse objectives of AI-governance structures: *first*, protection of established rights (i.e., privacy, and against discrimination, fraud, etc.); *second*, protection of established interests aiming to support economic growth, stimulate technological development, and promote the creation of novel markets and business models; and *third*, advancement of innovation with principles that seek to motivate AI research and development.^11^

Three core principles are emerging, which may inform future AI initiatives: *first*, *learning,* which compels institutions that take charge of AI governance of the need to safeguard that the regulations they propose and adopt are flexible to be able to adjust and adapt to evolving circumstances; *second*, *interoperability,* which permits different systems and data structures to communicate without aiming for total unification or harmonization, requiring information to be communicated in a structure recognized by both the sender and the receiver (known as syntactic interoperability), both of whom understand the meaning of the communicated information (known as semantic interoperability);^2^^,^^12^ *third*, *capacity-building,* to translate legal requirements to policies as well as best practices, technical principles, and standards into everyday usage cases; governance initiatives should invest in effective implementation of capacity-building, which considers AI literateness, technical support, and assistance,^11^ as well as access to digital technologies.

### The UN approach on AI

#### Policy

In March, 2024, the United Nations General Assembly (UNGA)^13^ adopted the U.S.-led resolution on AI, attempting to reach a worldwide agreement that would enable and advance the safe, secure, trustworthy, and sustainable AI development. The resolution only “encourages” Member States to foster the safe, secure, and trustworthy development of AI technologies by adopting and implementing a set of policies in order to protect individuals from misuse from harmful practices; thus, legally, it carries a more symbolic character than actual weight.

Furthermore, the repeated reaffirmation in Resolution 78/L.49 that Member States must only fulfill these policies in accordance with their national laws, allows for a high level of discretion for Member States regarding its implementation. Thus, Member States remain responsible for defining the needs and provisions for their country, and are not mandated to relinquish their regional and national policies, facilitating them to carry on pursuing their distinct interests.

The resolution helps ensure that the AI benefits reach all countries, and at all levels of development, and focuses on capacity building and bridging existing digital divides, especially in developing countries. It underscores the agreement that AI systems can both respect human rights and fundamental freedoms, as well as nurture and raise expectations for sustainable development and growth. It is expected that governments will work with all stakeholders, including civil society, the private sector, regional and international organizations, academic and research institutions as well as technical organizations, to collectively develop and build this framework. Importantly, this resolution is expected to serve as a foundation for multilateral AI efforts on current and future UN initiatives.

#### Enforcement

There is no single, legally binding “United Nations policy” document that directly regulates the use of artificial intelligence (AI) in healthcare for all UN Member States. The UN does not have the authority to create laws or binding regulations that directly govern the internal policies of sovereign nations in the way that a national government or the European Union can. In summary, the UN “enforces” its policies on AI in healthcare primarily by setting global ethical and normative standards, providing guidance and technical assistance, facilitating international dialog, and creating an environment of expectation and best practice that encourages member states to adopt and implement responsible AI policies within their own national jurisdictions. The actual legal enforcement occurs at the national level, informed and influenced by the UN's global frameworks.

### The United States Government's approach on AI

#### Policy

On October 30, 2023, the White House, in an attempt to institute a more unified federal policy on AI, issued an executive order, which directed the development of new governance structures, guidelines, and reports pertinent to AI.^14^ On March 28, 2024, President Biden introduced a new policy to strengthen the capabilities of federal agencies to leverage the benefits of AI technologies, identifying AI uses according to risk. The policy took a risk-based methodology to arrange funds and assets for AI accountability by placing less attention on low-risk AI technologies or those that can be administered by other policies, and focused instead on AI technologies that may well impact individual safety and/or rights.

Since January 2025, the Trump administration has reset federal AI policy in ways that directly shape health-care oversight and deployment, by unwinding the prior framework, rescinding Biden-era directives and replacing them with Executive Order 14,179, which aims to “remove barriers” to U.S. AI leadership and ordered a review/suspension of actions taken under EO 14110.^15^ The Office of Management and Budget then issued new, pro-innovation government-wide AI memoranda—M-25-21 (use/governance) and M-25-22 (acquisition)—that supersede M-24-10 and M-24-18 and instruct agencies (including HHS) to name Chief AI Officers, set generative-AI policies, and streamline procurement, changes that affect how AI tools are bought and used across federal health programs.^16^ The White House followed with “America's AI Action Plan,” a roadmap emphasizing deregulation, workforce development, and standards.

At the sector level, FDA finalized its guidance on Predetermined Change Control Plans (PCCPs), (Aug. 18, 2025), clarifying how AI-enabled medical devices can iterate safely post-market, an important enabler for SaMD updates in clinical care.^17^ Office of the National Coordinator's (ONC) HTI-1 rule, took effect in 2025 and remains a major transparency lever: “predictive decision support interventions” in certified EHRs must expose key facts about training data, logic, and performance for clinicians.^18^

#### Enforcement

Enforcement of regulations concerning AI in healthcare in the U.S. is multi-layered, decentralized, and depends significantly on the specific type of regulation, the agency involved [i.e. the Food and Drug Administration (FDA), Department of Health and Human Service (HHS) and the ONC, the National Institute of Standards and Technology (NIST), the Federal Trade Commission (FTC), and Congressional Action], and the nature and the risk of the AI application. Because there is not one single AI-specific law, enforcement is achieved through a combination of existing regulatory frameworks, agency-specific powers, and evolving approaches. Enforcement approaches are still evolving alongside AI technology. Agencies are adapting their methods to address the unique characteristics of AI (e.g., continuous learning, algorithmic complexity), with a focus on compliance and deterrence.

The HHS Office of Inspector General (OIG) has also pivoted to tougher enforcement against “information blocking,” by imposing civil monetary penalties of up to $1 million per violation, signaling more pressure on providers/EHRs to share data needed for AI-enabled care coordination.^19^ In parallel, CMS's July 2024 final rule creates provider “disincentives” when OIG determines information blocking—e.g., loss of “meaningful EHR user” status for hospitals/CAHs, a zero Promoting Interoperability score in MIPS for clinicians, and removal or denial of participation in the Medicare Shared Savings Program for at least one year. ONC's HTI-1 Final Rule adds enforceable transparency obligations for “decision support interventions” (including predictive/AI tools) within the Health IT Certification Program; ONC may conduct direct reviews and suspend or terminate certification for non-conformity.^20^ FDA enforces safety/effectiveness for AI-enabled devices and, with its final guidance on PCCPs, allows pre-authorized algorithm updates while keeping modifications outside an approved PCCP subject to additional review and potential enforcement under device laws. HHS-OCR's 2024 Section 1557 rule prohibits discrimination through the use of patient-care decision-support tools and requires covered entities to identify and mitigate bias—standards enforceable by OCR beginning in 2025.^21^ Finally, the FTC's 2024 amendments to the Health Breach Notification Rule extend breach-notice duties to many health apps; recent cases (e.g., GoodRx, BetterHelp) show the agency will penalize deceptive data uses and bar sharing sensitive health data for advertising.

### The United Kingdom Government's approach on AI

#### Policy

The United Kingdom's (UK) government has issued a white-paper promoting a pro-innovation approach, principally in commercial AI applications.^22^ The white-paper details how the UK government intends to support innovation in a framework that ensures that risks are recognized and addressed, recognizing that a strict or rigid approach can hinder innovation and slow AI research and development. Furthermore, the white-paper sets out a balanced and pro-innovation regulatory framework, which, instead of targeting specific technologies, provides some non-binding principles that focus on the context of the deployed AI technology, providing a sensible approach in balancing the benefits versus the potential risks.

However, the emphasis on supporting commercial innovation has invited criticism for under-appreciating the growing usage of AI in UK government sectors, such as healthcare and education.

#### Enforcement

Enforcement is multi-layered, and favors a risk-based and proportionate approach, combining legally binding regulations [Medicines and Healthcare products Regulatory Agency (MHRA), Information Commissioner's Office (ICO)], system-level influence (NHS England commissioning, and professional/ethical self-regulation. It seeks a balance between formal rules and professional judgment. Specifically, the: (i) MHRA regulatory oversight: For AI used as or in medical devices, the MHRA's pre-market and post-market regulatory oversight is a key enforcement mechanism. Failure to comply with MHRA regulations can lead to devices being removed from the market and legal penalties; (ii) ICO data protection enforcement: The ICO's enforcement powers under UK GDPR and the Data Protection Act are significant. Fines, enforcement notices, and reputational damage are deterrents for data protection violations; (iii) NHS Commissioning and Contracting: NHS England's guidance and standards can be enforced through commissioning contracts and procurement processes. NHS organizations are incentivized to adopt compliant AI solutions to receive funding and operate effectively. In summary, the UK government *does* have a policy framework enforcing AI regulations in healthcare. It is a comprehensive, evolving, and multi-faceted system composed of national strategies, regulatory adaptations by the MHRA, ethical guidelines, operational guidance from NHS England, and data protection enforcement by the ICO.

### The European Union's approach on AI

#### Policy

Recently, the EU passed a landmark legislation on AI (EU Act).^23^ The EU Act has been the first significant attempt to provide a comprehensive legal framework in the world that aimed to regulate and administer the ever growing AI technology ecosystem. The legislation for the first time intends to safeguard the diverse aspects of fundamental human rights, the rule of law, democratic governance and environmental sustainability, from the impact of high-risk AI, while simultaneously encourages and supports the AI technological innovation. Thus, according to the Act, while the essential and practical requirements increase as a pyramid of risks, they have to be accompanied with particularly strong safeguards as the risks of the AI systems increase. Furthermore, the EU Act details requirements that AI developers must meet before placing their products on the EU markets. Finally, the EU Act, which is enhanced by sector-specific regulations, puts forward a multi-faceted oversight structure to monitor compliance and enforce regulations with the threat of substantial fines.

However, the Act comes with risks, as well: *First*, the wide-ranging designations and classifications within the Act present challenges, and create likely ambiguity for AI developers and users. *Second*, the stringent regulations are likely to place EU companies at a competitive disadvantage worldwide, in particular against those companies located in countries with lighter AI regulations.

Despite the pros and cons, there is a legitimate debate regarding whether the Act has kept pace with the evolving AI technologies. For example, the Act necessitates a sufficiently comprehensive report of the data used to train AI models of general purpose, as well as guards for trade secrets; yet, many AI companies are not eager to disclose details regarding how they train their models, and, therefore, they may look to by-pass these regulations by pursuing research and development in less regulatory restricting countries. Such a scenario may produce a race to an unknown bottom situation, in which countries interested in winning the AI race are therefore encouraged or prone to adopt lower-level regulations and accountability.

Finally, harm-mitigation measures should be placed to ensure that people who experience harm from AI-based as well as other digital technologies receive compensation. Finally, very little progress has been made with respect to the EU cooperation at the international global level.

#### Enforcement

In summary, enforcement of EU regulations for AI in healthcare is robust and multi-faceted, involving pre-market controls, market surveillance, data protection monitoring, corrective actions, and significant financial penalties. National Competent Authorities and Data Protection Authorities in each member state are the key enforcers, with coordination and consistency efforts at the EU level. The framework is designed to be comprehensive, to be risk-based, and to promote responsible and ethical AI innovation in healthcare while safeguarding patient safety, data privacy, and fundamental rights.

### The Indian Government's approach on AI

#### Policy

In the advent of AI-governing legislation around the world, it has been inevitable its impact to be felt globally, including in India. On March 15, 2024,^24^ the Indian Government, using an updated AI policy advisory issued by the Ministry of Electronics and IT, addressed the risk in AI driven technologies.

With an exception for start-up companies, the policy advisory has made an effort to foster the transformational capacity of AI by enabling the innovative technological services and capabilities of the country, while respecting the rights of the citizens of the country to be informed of possible risks. The focus is on leveraging AI to improve healthcare access, affordability, and quality, while also ensuring responsible and ethical development and deployment in this sensitive sector.

#### Enforcement

Enforcement mechanisms are still evolving specifically for AI in healthcare, but rely on existing structures and advancing approaches. The Central Drugs Standard Control Organisation (CDSCO) is the most directly involved agency in enforcement concerning AI used *as* medical devices. Their enforcement powers are derived from the Drugs and Cosmetics Act, 1940 and the Medical Devices Rules, 2017. General laws like the IT Act and Consumer Protection Act can be invoked to address issues arising from misuse or harmful applications of AI in healthcare, particularly concerning data security breaches or misleading claims. In the initial phases, emphasis is also being placed on encouraging voluntary adoption of ethical guidelines and best practices by the industry. Industry associations and standards bodies can play a role in promoting self-regulation. In summary, India is actively building a policy ecosystem for regulating AI in healthcare. While a single, dedicated “AI in Healthcare Policy” law is not yet in place, a framework is emerging through national strategies, adaptation of existing regulations (especially medical device rules and data protection law), and the development of ethical guidelines.

### The Chinese Government's approach in AI

#### Policy

The Chinese government has put forward an aspiring strategy, outlined in the initiative, “New Generation Artificial Intelligence Development Plan,”^25^ which aims to establish China as a world front-runner in AI innovation by 2030. With respect to putting forward regulatory policies, China has introduced and adopted many regulatory guidelines and standards that focus on national security, data protection, and algorithmic transparency; however, there exist concerns with respect to the central government's engagement in surveillance, restriction, and suppression practices that employ AI technologies.

#### Enforcement

Enforcement of regulations concerning AI in healthcare in China is multi-layered, strongly driven by the central government, with many agencies playing leading roles [i.e. the National Medical Products Administration (NMPA), and the Cyberspace Administration of China (CAC), the National Health Commission (NHC), the Ministry of Industry and Information Technology (MIIT) and the National Development and Reform Commission (NDRC)]. It is becoming more robust, with a shift towards clearer regulations, stronger penalties (especially financial under the Personal Information Protection Law), and more active monitoring. It operates through a combination of administrative actions, legal penalties, industry oversight, and “soft law” mechanisms. Enforcement varies depending on the specific regulation, the agency involved, and the nature of the AI application.

### The Canadian Government's approach in AI

#### Policy

Canada's official policy on AI in health care is anchored in Health Canada's regulation of AI/ML-enabled medical devices under the *Food and Drugs Act* and *Medical Devices Regulations*, which treat ML-enabled medical devices (MLMDs) as medical devices subject to safety and effectiveness requirements across the product lifecycle.^26^ Foundational classification is set by the SaMD guidance, which clarifies inclusion/exclusion criteria and risk classes for software used for diagnosis, treatment, monitoring, or decision support.^26^ In 2025, Health Canada issued Pre-market Guidance for MLMDs introducing PCCPs so certain model updates can be pre-authorized, and detailing expectations for evidence, transparency, post-market monitoring and sex- and gender-based analysis plus, to support equitable performance.^26^ Complementing device-specific rules, Canada (in coordination with FDA and MHRA) has published Good Machine Learning Practice (GMLP) guiding principles and Transparency guiding principles for MLMDs, emphasizing human-AI team performance, explainability, user-appropriate information, and lifecycle oversight.^27^ At the health-system level, federal, provincial and territorial ministers (except Quebec, which maintains autonomy) endorsed Pan-Canadian AI for Health (AI4H) Guiding Principles that commit to person-centricity, equity, privacy and security, safety and oversight, accountability, transparency, AI literacy, robust data practices, and Indigenous-led governance and data sovereignty for AI adoption across Canada's health systems.^26^ Together, these guidance, classification, MLMD pre-market guidance with PCCPs, GMLP and transparency principles, and the AI4H system-level values—constitute the Government of Canada's official policy framework for responsible, risk-based AI in health care.^28^

#### Enforcement

Canada's AI-in-health-care enforcement operates through Health Canada's medical device regime: AI/ML-enabled systems used for diagnosis, treatment, monitoring or decision support are enforced under the Food and Drugs Act *and the* Medical Devices Regulations using a risk-based compliance and enforcement (C&E) framework (inspections, stop-sale, license suspension/cancellation, recalls).^27^ For medical-device establishments, Health Canada conducts inspections and can impose terms and conditions, require corrective action within tight timelines, and suspend or refuse licenses where risks persist.^27^ Post-market, regulated parties must keep distribution/complaint records to enable rapid, effective recalls, and hospitals/manufacturers must report serious medical device incidents within prescribed time frames; non-compliance can trigger escalated enforcement.^29^ Health Canada also wields statutory recall powers: the Minister may order recalls or corrective actions when a device poses a serious or imminent risk, guided by the federal Recall Policy (POL-0016).^30^ For AI/ML devices specifically, pre-market guidance for ML-enabled medical devices (including PCCPs) sets lifecycle obligations for monitoring, evidence, and transparency; failure to meet authorized change protocols or reporting duties is addressed via the same C&E tools (letters, stop-sale, license actions, recalls).^27^ Mandatory problem reporting requirements for manufacturers/importers further underpin enforcement, with Health Canada forms and guidance specifying what must be reported and when.^26^ In short, Canada enforces AI in health care by applying its medical-device laws, inspection-driven, risk-proportionate actions backed by recall orders and recordkeeping/reporting duties, rather than a separate AI-specific enforcement statute.^26^

### The Japan Government's approach in AI

#### Policy

Japan's official policy for AI in health care couples pro-innovation with patient safety under the Pharmaceuticals and Medical Devices Act (PMDA), which has regulated SaMD since November 25, 2014, with the PMDA as the reviewing authority and International Medical Device Regulators Forum (IMDRF) concepts informing terminology and evaluation.^31^ To make review predictable, the Ministry of Health, Labor and Welfare (MHLW) issued the 2019 “Guidance for evaluation of artificial intelligence–assisted medical imaging systems for clinical diagnosis” (Annex 4, No. 2), setting out efficacy, safety, and clinical-performance considerations for AI systems.^32^ Japan also adapted regulation for learning systems by introducing a Post-Approval Change Management Protocol (PACMP) in 2020, enabling pre-specified, lifecycle updates to AI/ML SaMD while maintaining oversight.^33^ PMDA's Science Board has since consolidated regulatory science issues for AI-based SaMD and highlighted GMLP principles across data quality, validation, and post-market monitoring, further operationalizing review expectations.^33^ On data governance, the Next-Generation Medical Infrastructure Act (2017; in force 2018) authorizes the secondary use of medical information for R&D through certified processors under strict anonymization/pseudonymization safeguards, with 2024 amendments expanding the use of pseudonymized electronic health record data to accelerate AI development.^34^ Deployment is promoted via the Cabinet Office's Strategic Innovation Promotion Program “AI Hospital System,” which funds clinical pathways and infrastructure for safe AI uptake, reinforced by professional implementation support.^35^ Overall, Japan's health-AI policy blends device-centric regulation (PMDA/MHLW guidance, PACMP) with privacy-preserving data access and targeted innovation programs to align safety, effectiveness, and real-world adoption.^35^^,^^36^

#### Enforcement

Japan enforces health-care AI primarily through the PMDA, applying the existing medical-device regime to AI/ML software used for diagnosis or treatment. Premarket enforcement runs through PMDA's scientific review under MHLW authority, with good-practice inspections validating data integrity and compliance.^33^ Once on the market, Marketing Authorization Holders must report adverse events and recalls (Articles 68–10 and 68–11), while MHLW/PMDA can conduct on-site inspections and issue emergency or improvement orders (Chapter XI & XIII).^37^ Criminal penalties also backstop non-compliance (Chapter XVII “Penalties”).^37^ MHLW's AI imaging guidance operationalizes review expectations (validation, clinical performance), providing a concrete basis for regulatory decisions and post-market controls. Data-use enforcement sits alongside device rules: under the Next-Generation Medical Infrastructure Act, only certified entities may create anonymized medical data; they must implement security controls, are subject to revocation of certification for violations, and the statute contains penal provisions.^38^ PMDA also specifies practical reporting mechanics for Marketing Authorization Holders and health professionals, operationalizing Article 68-10 duties.^31^ In short, Japan's health-AI enforcement is not a separate AI code but a robust device-and-data framework: premarket review, mandatory vigilance/recall reporting, inspections and orders, lifecycle control of ML changes, certified data governance, and criminal/administrative sanctions when needed.^38^

## Divergent interests and policies towards a yet to be decided common goal

As the DoH undertakes the task of addressing a complex global ethical landscape, ongoing conflict, controversy, and debate are inevitable. In an environment of rapid growth of AI algorithms and technologies that has urged many governments to begin developing regulatory policies, the 2024 DoH revision represents a meaningful renewal of this ethical document that promotes and ensures respect for and protection of the rights and interests of all medical research participants.

In the US, lack of a central policy framework can lead to ambiguities that may potentially create a more complex fragmented framework of regulations for AI companies. Consequently, without a unified national strategy compared to EU or the UK, the US may fall behind in promoting worldwide AI governing standards. By contrast, the new UN resolution (78/L.49),^13^ while non-binding, is a first attempt to get all countries on board recognizing the extensively divergent interests, not only from continent to continent, but from country to country, as well. While the EU and the US, collaboratively and complementarily, are supporting the potential of AI to drive research and development in order to address global challenges,^39^ African States struggle mostly with accessibility and participation in AI advancements, where AI-driven technologies are highly dependent on internet availability (the majority of the 33% of the world's population that is lacking internet access lives in the Global South).

From a governance perspective, policy diversity reflects the fact that governments and agencies that institute regulatory policies have divergent philosophies and priorities, and, therefore, many other national and international governance initiatives form a complex and thickening landscape of intertwined principles, norms, processes, and institutions influencing the development and use of AI (Box 3).

Box 3 **Healthcare AI policy recommendations to address the 2024 revisions of the DoH.**

**Table:** 

• Respect, protect, and foster human rights and freedoms throughout the life-cycle of AI technologies • Protect individuals from all forms of discrimination, bias, misuse, or other potential harms resulting from AI technologies • Develop policy and governance approaches of AI technologies that require proper testing of these technologies prior to their deployment, and that guarantee their robustness, transparency, explainability, accountability, contestability, and redress • Cooperate in reducing the digital and AI divides within and between countries and continents by fostering and enabling equitable access to the benefits of AI technologies • Safeguard the privacy and protection of personal data in using AI technologies • Improve education and raise public awareness of the appropriate citizen use of AI technologies • Provide capacity building as well as financial and technical assistance to developing countries • Deter and mitigate potential negative consequences of the use of AI technologies • Respect intellectual property rights of sectors researching and developing AI technologies • Encourage and promote the development of technologies that determine AI-generated digital content • Encourage the private sector of AI technologies to adhere to domestic and international laws and regulations

Interestingly, despite the growing prominence of AI in clinical research and health care, the recently revised DoH and the absence of detailed guidance in confronting many emerging ethical dilemmas, including issues of data privacy^10^ and bias,^40^ AI algorithm bias, and the broader role of AI in clinical decision making towards encoding equity, was notable.^1^

## Implicit mandates of the DoH on AI governance

An AI related ethical principle is a living, ethical statement of a responsibility pertaining to the development, deployment, and assessment throughout its lifecycle of AI-enabled health technologies, applied to all persons, pertinent to:

• Avoiding harming others (or “non-maleficence”).
• Promoting the well-being of others (or “beneficence”), while minimizing risks of harm, or balancing them against expected benefits.
• Ensuring that all persons are treated fairly (“justice” or “fairness”).
• Respecting persons' interests in making decisions about their lives (or “autonomy”).
• Protecting individual privacy and self-determination (respect to autonomy).

Given that ethical principles do not always clearly discuss limitations regarding the uses of technologies, governments should limit the use of AI-enabled health technologies should they violate or endanger human rights, do not conform to established principles or regulations or are introduced prematurely or other non-relevant to the intended-use contexts.

Ethical principles and human rights are closely linked. Because human rights are legally binding, they provide a powerful framework by which private sector stakeholders, international organizations and governments, are obligated to abide. However, the existence of a human rights framework does not obviate the necessity for continuing deliberations on ethical matters, that aim to expand and complement norms and obligations established by human rights declarations. In many circumstances, multiple ethical considerations become relevant which require appropriate incorporation in order to ensure that the different perspectives are included, and establish a decision-making process that the involved stakeholders consider fair and with outcomes that are considered legitimate.

These ethical principles are intended to provide education of those who develop and use such technologies, and guidance to all stakeholders (policy-makers, local and national governments, health system developers, health organizations, administrators and clinicians) about how basic ethical requirements should direct or constrain their decisions and actions during the development, assessment of their performance and the deployment of AI-enabled health technologies.^2^^,^^9^^,^^41^, ^42^, ^43^

Therefore, ethical principles should also be linked to professional and technological standards for AI-enabled health technologies. Given that these technologies, incrementally, have no-borders, private sector stakeholders have the responsibility to respect human rights, independently of their governmental obligations. Therefore, in satisfying this responsibility, private sector stakeholders must take continuous proactive and reactive steps to ensure that they do not mistreat or contribute to the mistreatment of human rights. Recently, the appearance of non-health dedicated commercial entities into health care, a form of ‘sphere transgression’^44^ that expands their business models, may place commercial priorities over patient rights, therefore undermining trust, accountability, and transparency, while monopolizing the acquired data without public scrutiny, citing trade secrets and proprietary AI, demanding self-regulation, and limiting independent validation of fairness, as well as mitigation of bias.

## Conclusions

The workgroup assigned to develop the 2024 revisions of the DoH^1^ recognized a lack of adequate consideration to consent requirements and protection of participants, related to the growing use and associated risks of stored personal data following clinical research trials, and, in particular, in the context of the emergence of AI, genetic data collection, capacity to re-identify de-identified data, and the potential commercial and/or political misuse of these data. Implementation of the DoH today requires a dynamic and adaptive international governance framework, overseen by a regulatory body that will establish global AI policies that will be responsible for monitoring and advancing its regulations in pace with the turbulent growth of AI technologies.

Because HIPAA is entity- and transaction-scoped, not all commercial entities of health-related data are covered; our recommendations therefore pair sectoral health rules with general AI and data-protection safeguards to avoid gaps.

## Contributors

AAA contributed to the conception and writing of the manuscript.

JL contributed to the conception and writing of the manuscript.

All authors have read and approved the final version of the manuscript.

## Declaration of interests

AAA has nothing to declare.

JL consults for 5 AM Ventures, has stock options in Ionis, Inc. and Leap Therapeutics, Inc., and has financial or non-financial interests in Scipher Medicine, Inc., Precede Biosciences, Inc.
